# Supplementary material for: Impact of mid Eocene greenhouse warming on America’s southernmost floras
Source: Commun Biol. 2021 Feb 9;4:176. doi: 10.1038/s42003-021-01701-5 (PMC7873257; doi:10.1038/s42003-021-01701-5)
Supplement: Supplementary file 2 — Supplementary Information [file 42003_2021_1701_MOESM2_ESM.pdf]

Impact of mid Eocene greenhouse warming on America's southernmost floras

Fernández et al.

Table of Contents

Supplementary Note 1: Stratigraphy and previous paleobotanical studies

Supplementary Note 2: Age model based on dinocyst assemblages

Supplementary Note 3: Spore-pollen assemblages

Supplementary Note 4: Selected fossil forms with tropical and subtropical affinity

Supplementary Note 5: R scripts

Supplementary References

Supplementary Figure 1. Schematic correlation of the Río Turbio Formation showing sections and sample locations.

Supplementary Figure 2. Selected dinocysts species from the Eocene of southern South America.

Supplementary Figure 3. Trends in dinocysts frequency.

Supplementary Figure 4. Quantitative distribution of the dinocyst assemblages from the Río Turbio Formation.

Supplementary Figure 5. Dinocyst events and zones recorded in the Río Turbio Formation.

Supplementary Figure 6. Fossil forms with tropical and subtropical affinity from the RTF.

Supplementary Information

Impact of mid Eocene greenhouse warming on America's southernmost floras

Supplementary Note 1: Stratigraphy and previous paleobotanical studies

The Río Turbio Formation (1–2) comprises approximately 550–600 m of marine, transitional and terrestrial sediments, representing shallow-marine and estuarine successions. Two stratigraphically distinct (informal) members were recognized; a lower member (LM) made up of about 290 m of conglomerates and coarse- to medium-grained sandstones, assigned to the early to middle Eocene (3–4) and an upper member (UM) of 300 m of fine to coarse sandstones and conglomerates with interbedded clay horizons, representing coastal marine, wave- and tide-dominated environments, assigned to middle to late Eocene (5). Most of the samples collected for the present study comes from the UM (Supplementary Fig. 4). Both the LM and UM contain coal seams of up to 2 m thick with abundant fossil plant remains. The Río Turbio Formation conformably overlies the late Cretaceous Cerro Dorotea Formation (3, 5) and it is unconformably overlain by the Miocene Río Guillermo Formation (6–8). Fossil remains either of plants (leaves and woods) and spore-pollen and dinocyst assemblages preserved at the Río Turbio Formation have been studied in detail since the last several decades (9–22). Although the diversity estimates have never been quantified so far for the Río Turbio Formation on the basis of the spore-pollen record, these studies constrained the age of the sedimentary unit, and provided a general picture of what landscapes looked like during the Paleogene in southern South America.

Supplementary Note 2: Age model based on dinocyst assemblages

The dinocyst stratigraphy of the Río Turbio Formation is largely based on previous zonation schemes (RTF 20–22). These schemes, along with others from Australasia (e.g. 23),

are here used to constrain the extension of the MECO using our recovered dinocyst specimens. Overall, the Río Turbio Formation preserves poorly diverse dinocyst assemblages, mostly composed by endemic Antarctic (e.g. *Enneadocysta dictyostila*, *Vozzhennikovia apertura*, *Deflandrea antarctica*) and cosmopolitan (e.g. *Turbiosphaera filosa*, *Operculodinium* spp. and *Spiniferites* spp.) taxa, with shifts in abundance across the composite section (Supplementary Fig. 2-4). The dinocyst species preserved in our selected 33 samples are assembled within three major intervals: Interval A (samples 1 to 7), Interval B (samples 8 to 25) and Interval C (samples 26 to 32) according to our constrained cluster analysis (Supplementary Fig. 3).

Interval **A**) includes scarce *Enneadocysta dictyostila* (up to 10 %), and peaks of both *Vozzhennikovia apertura* and *Deflandrea antarctica*. This interval A partially coincides with Sequences I to III defined by Rodríguez Raising (24) for the Río Turbio Fm, recently dated to the mid Eocene (47.1 and 46.3 Myr) based on zircon U-Pb ages near the Cancha Carrera area (25). Our dinocyst assemblage of Interval A correlates with the Río Turbio Dinocyst Zone (RTF) 1 (~46 Myr, 22) and with the Southern Pacific Dinocyst Zone (SPDZ) 10 (middle Lutetian, 46.2– 45.2 Myr; 26).

Interval **B**) includes peaks in relative abundances of *Enneadocysta dictyostila* (up to 95%) associated with *Hystrichosphaeridium truswelliae* and *Arachnodinium antarcticum*. Our interval B coincides with Sequences IV to VI of Rodríguez Raising (24), that contains within Sequence VI a thick glauconitic level associated with a Maximum Flooding Surface (MFS). Recently Fosdick et al. (25) dated this glauconitic level in the Cancha Carrera area to the mid Eocene (41.3 Myr). This level was correlated with a Maximum Flooding Surface (MFS) in the Highway 40 sections by Rodríguez Raising (24). We identified this MFS within Interval B (between samples 14-15) based on a closed stratigraphical and palynological (dinocysts composition and abundances) comparison between our section and those of

Rodríguez Raising (24) and González Estebenet et al (20, 22). The upward samples of this interval continue typically characterized by peak abundances of *E. dictyostila*, also identified elsewhere in the Austral Basin (i.e. upper part of the equivalent Man Aike Formation (22, 27), Antarctic Peninsula (28) and South Tasman Rise ODP Site 1170 (23). Dinocyst assemblages from our Interval B correlates with the RTF 2 (~46 Myr – 39 Myr; 22) and with SPDZ 12 (44 Myr – 40 Myr; 20) and the base of the Zone SPDZ13 (40.0 Myr–35.95 Myr; 20).

Interval **C**) includes peaks of *Vozzhennikovia apertura* and *Turbiosphaera filose* (Supplementary Fig. 4). Our interval C is equivalent to Sequences VII to IX of Rodríguez Raising (24). The lowest common occurrences of *Turbiosphaera filosa* (LCO: ~35.5 Myr; 21) together with the HOs of *Turbiosphaera filosa*, *Spinidinium macmurdoense* and *Vozzhennikovia* spp. (HOs: ~33.5 Myr; 21, 29) allow us to assign this interval to the middle to late Priabonian age (~35.5 Myr –33.5 Myr; 30). Recently, U-Pb geochronological results from the Río Turbio Formation in the Cancha Carrera area constrained the age of Sequences VIII between ~36.6–35.4 Myr and top of Sequence IX to ~26.6 Myr (25). Assemblages from our Interval C correlates with dinocyst zone RTF 4 of the Río Turbio Formation (~35.5–33.5 Myr; 30) and the Dinocyst Association (DA) 2 of the South Pacific Ocean (~35.5–33.5 Myr; 21).Towards the top of Interval C, typical endemic Antarctic assemblages were gradually replaced by more cosmopolitan ones, reflecting changes in the oceanographic and paleoenvironmental conditions relative to the Tasman Gateway deepening (~35.5 Myr; 21, 31–32) and the Sea of Hoces opening, also known as Drake Passage (33).

Supplementary Note 3: Spore-pollen assemblages

Our collected samples yielded moderate to large numbers of spores, pollen and dinocysts, in a matrix of well-preserved to carbonized plant debris (including cuticles), finely disseminated

charcoal particles and amorphous algal and fungal remains. The material recovered is relatively well-preserved, making identification at morphospecies level possible for most of the spore-pollen and dinocyst elements. The presence of tetrads (e.g. *Ericipites* sp. 1, *Bysmapollis verrucatus*) and clusters of pollen grains (e.g. *Nothofagidites*) suggest low-energy conditions at the time of the accumulation of the RTF.

Overall, the terrestrial palynological assemblages from RTF are dominated by sporomorphs (~60 %), with abundant dinocysts (~40 %) and some acritarchs and acari claws. The sporomorph assemblage consists of abundant angiosperm pollen (25–80 %) and ferns spores (10–50 %), with gymnosperm pollen as a minor component of the assemblage (10%). Angiosperm pollen grains are dominated by *Nothofagidites* spp. (mainly *Nothofagus* sg. *Nothofagus*) and *Myrtaceidites* spp. (Myrtaceae, Myrtoideae), with *Granodiporites nebulosus*, *Proteacidites* spp. and *Propylipollis* spp. (Proteaceae) as minor elements. Ferns spores are mainly represented by tree ferns (Cyatheaceae/Dicksoniaceae and Osmundaceae) which consist mainly of *Cyathidites minor* and *Trilites* spp. and to a lesser extent, of *Cyatheacidites annulatus* and *Baculatisporites* spp. Saccate pollen grains are mainly represented by *Podocarpidites* spp. and *Phyllocladidites mawsonii* (Podocarpaceae); another gymnosperm is *Araucariacites australis* (Araucariaceae, Araucaria). Furthermore, occurrences of tropical species, *Ilexpollenites* spp. (Aquifoliaceae, *Ilex*), *Cupaneidites* spp. (Sapindaceae, *Cupania*), *Bombacacidites isoreticulatus* (Malvaceae Bombacoidea), and five species of palms (Arecaeae) are recorded (Fig. 6; Supplementary Fig. 6; Supplementary Data 2). At the beginning of the MECO ferns increase from ~30% to ~60% while angiosperms decrease dramatically from ~70% to ~30%. Also Podocarpaceae increases from ~5 % to ~20% and tropical groups from ~2 % to ~6%. At the core of the MECO ferns drop to a minimum, while angiosperms become dominant (80%). Apart from the dominant lineages (i.e. *Nothofagus*, Podocarpaceae and Dicksoniaceae), other gondwanan families (e.g.

Myrtaceae and Proteaceae) became important elements (Fig. 8). Tropical lineages also became common and diverse (~85%) across the MECO. At the end of the MECO (sub-group 3, samples 22–25) ferns rise again to maximum values (ca. 60%) while angiosperms and tropical groups decrease at the same time. Post-MECO ferns decrease towards the top while Podocarpaceae remains abundant and a drop of tropical forms diversity (~40%) is recorded (Fig. 8).

Supplementary Note 4: Selected fossil forms with tropical and subtropical affinity

Previous records, botanical affinity and geographical distribution of living representatives of specimens with tropical and subtropical affinity are given.

Ferns

Biretisporites crassilabratus Archangelsky, 1972. **Supplementary Figure 6. 1**

This species has been widely recorded both in Argentina (16, 35–40) and in Brazil (41–43) during warm periods of the Paleogene and early Neogene.

Botanical affinity: This morphotype is comparable to those of family *Hymenophyllaceae* by having trilete laesura, psilate exospore and laesura as long as the longest diameter, crassimarginate (44–45). Similar morphological features are present in several species of *Hymenophyllum* (e.g. *H. brassii*, *H. kuhnii*) (46–47). Although species of this genus can reach southernmost regions of South America, the cold-adapted species (*Hymenophyllum caespitosum* Gaudich., *H. darwinii* Hook. f. ex Bosch, *H. dentatum* Cav., *H. falklandicum* Baker, *H. ferrugineum* Colla, *H. magellanicum* (Desv.) Willd. ex Kunze, *H. nahuelhuapiense* Diem & J.S. Licht., *H. pectinatum* Cav., *H. peltatum* (Poir.) Desv., *H. secundum* Hook. & Grev., *H. tortuosum* Hook. & Grev.) differ from the fossil *B. crassilabratus* by having heteromorphic papillaceous, granulated or coarsely equinate ornamentation (48).

Geographical distribution of living representatives: Tropics and, less commonly, subtropics, worldwide. Most species are either terrestrial or epiphytic, but a few are hemiepiphytes or lianas (49).

Distribution in Río Turbio Fm: Samples 1–2; 5–7; 11–13; 16–19; 21–22; 24–25; 31.

Cicatricosisporites sp. 4 Archangelsky et al., 1983. **Supplementary Figure 6.2**

This species and related forms have been widely recorded in Argentina (39, 50–51) and Colombia (52–53). This fossil species is commonly associated with other warm-demanding taxa (e.g. Arecaceae, Bombacoidea, *Cupania*, *Ilex*, Juglandaceae).

Botanical affinity: This morphotype is comparable to those of family Anemiaceae (formerly part of Schizaeaceae) by having trilete laesura and exospore canaliculate with irregular ribs (54–56). Similar morphological features are present in some modern spores of *Anemia* Swartz (e.g., *A. jaliscana* Maxon and *A. dregeana* Kunze) (54).

Geographical distribution of living representatives: Primarily neotropical. Only a few species in Africa-Madagascar and one in south India and islands in the Indian Ocean (49).

Distribution in Río Turbio Fm: Sample 11 (belonging to the MECO interval).

Crassoretitriletes vanraadshooveni Germeraad, Hopping y Müller, 1968. **Supplementary Figure 6.3**

This fossil species has been widely recorded both in northern South America (57–60) and in Australia (e.g. 61–63) during periods with a humid tropical or equatorial climate in the Paleogene and Neogene.

Botanical affinity: This morphotype is comparable to those of genus *Lygodium* (Lygodiaceae). It is trilete laesura, medium-large size (50 μm or more), with the sporoderm composed of perispore and exospore. Surface sculpture of exospore ranges from smooth to

scabrate, verrucate, rugulate, or reticulate (64). The spores found in the RTF have perispore thin, scabrate and exospore rugulo-reticulate.

Geographical distribution of living representatives: *Lygodium* species are mainly found in tropical regions; there are only a few temperate outliers in North America and Japan (65–66). Most living species prefer moist environments, although a few are found in areas with a pronounced dry season (67).

Distribution in Río Turbio Fm: Samples 8–14; 22–23; 25–26; 32.

Klukisporites scaberis (Cookson y Dettmann, 1958) Dettmann, 1963. **Supplementary Figure 6.4**

This fossil species has been widely recorded both in Brazil (42) and in Australia (68 and references there in) during warm periods of the late Cretaceous and Paleogene.

Botanical affinity: This morphotype is comparable to those of family Schizaceae by having trilete laesura and foveo-reticulate pole (54, 69–73). Similar morphological features are present microspores found in the schizaeaceous species *Klukiaexilis* (Phillips) and *Stachypteris hallei* Thomas. Comparable spores have been described by Harris, T. M. (74, p. 137) from the closely related species *S. spicans* Pomel. (54).

Geographical distribution of living representatives: Tropical and warm-temperate regions worldwide. Many species are characteristic of sandy soils (49).

Distribution in Río Turbio Fm: Sample 10; 15; 25 (all belonging to the MECO interval).

Kuylisporites waterbolkii Potonié, 1956. **Supplementary Figure 6.5**

Fossil species commonly found in South America (57,75–78) and in Australia (e.g. 62–63, 79–81). This fossil species is commonly associated with other meso-megatermic taxa (e.g. Arecaceae, Bombacoidea, *Cupania*).

Botanical affinity: This morphotype is comparable to those of genus *Cnemidaria* sp. (Cyatheacea) by having trilete laesura and three large pits (diameter about 5-7 µm) symmetrically arranged at the center of the three sides (82).

Geographical distribution of living representatives: The geographic range of living *Cnemidaria* covers the area of Central America, the Greater Antilles, and the northern part of South America (82).

Distribution in Río Turbio Fm: Samples 9 and 20 (all belonging to the MECO interval).

Dicots

Anacolosidites diffusa Archangelsky 1973. **Supplementary Figure 6.6**

Fossil species commonly found in Argentina during warm peaks of the late Cretaceous to Paleocene (83–87).

Botanical affinity: The fossil species presents general similarities with the pollen types recognized in *Cathedra* (Olacaceae) by having amb rounded-triangular, tri-diporate (i.e. the apertures comprise three pairs of pores, and the two members of each pair face each other on opposite hemispheres, without any suggestion of an ectocolpus), pore elliptic close to the corner of the grain in polar view, exine scabrate (88).

Geographical distribution of living representatives: Species of the genus *Cathedra* are exclusively neotropical. Among the five recognised species, only one is known from wet forests, the other ones are recorded from dryer ecosystems (restinga) (88).

Distribution in Río Turbio Fm: Sample 30.

Bombacacidites isoreticulatus McIntyre 1965. **Figure 8.9**

Fossil species found in Argentina (39) and New Zealand during warm peaks of the Paleogene (89–92).

Botanical affinity: These morphotypes are comparable to those of genus *Ceiba* sp. (Malvaceae, Bombacoidea) by having amb circular, tricolporate aperture with ectoaperture short, narrow and endoaperture circular; exine reticulate more or less homobrochate, muri less than 1 µm wide, simplibaculate.

Geographical distribution of living representatives: In southern South America *Ceiba* is represented by four species restricted to subtropical areas. *C. speciosa* (A. St.-Hil.) Ravenna is the southernmost species, growing at warm-temperate latitudes, reaching 29ºS, in central Argentina (93).

Distribution in Río Turbio Fm: Samples 9–12; 14; 17; 21 (all belonging to the MECO interval).

Cupanieidites insularis Mildenhall &Pocknall 1989. **Figure 8.10**

Fossil species found in early to middle Miocene of New Zealand (94).

Botanical affinity: The analyzed fossil pollen presents general similarities with the pollen of *Cupania* (Sapindaceae), both types are isopolar, whit3-syncolporate aperture, triangular outline in polar view, peroblate.

Geographical distribution of living representatives: *Cupania*is distributed mainly in tropical regions (95). In southern South America, *Cupaniavernalis* Cambess. is the southernmost species of the genus, growing in northeastern (30ºS), Argentina (93).

Distribution in Río Turbio Fm: Samples 4; 14; 19; 21; 25; 30.

Cupanieidites reticularis Cookson & Pike 1954. **Supplementary Figure 6.7**

This fossil species has been widely recorded both in Argentina (96–100) and in Brazil (41, 57, 101–103) during warm periods of the late Cretaceous to early Neogene.

Botanical affinity: The fossil pollen is similar to that of *Cardiospermum grandiflorum* (Sapindaceae). Both types are heteropolar, 3-syncolporate, with subtriangular or triangular outline in polar view; exine microreticulate (104).

Geographical distribution of living representatives: In southern South America, *Cardiospermum grandiflorum* is the southernmost species of Sapindaceae, growing in the northern Buenos Aires province, Argentina (93).

Distribution in Río TurbioFm: Samples 9; 14; 19 (all belonging to the MECO interval).

*Ilexpollenites anguloclavatus*McIntyre 1968; *I. clifdenensis*McIntyre 1968; *I. megagemmatus* McIntyre 1968. **Figure 8.7; Supplementary Figure 6.8–9**

This fossil species has been widely recorded both in Argentina (105–107) and in Australia (63, 108–116) during periods of the late Cretaceus to early Neogene when tropical climates prevailed.

Botanical affinity: These morphotypes are comparable to those of genus *Ilex* sp. (Aquifoliaceae) by having tricolporate apertures with poorly defined pores, with conspicuous gemmae and clavae of variable size (79).

Geographical distribution of living representatives: Most species have their home in the tropics and subtropics, with a wide distribution in temperate areas of Asia, Europe, Africa (a single species), North and South America, where endemisms are prominent (117–118). In Argentina, *Ilex* is restricted to subtropical areas (93).

Distribution in Río Turbio Fm: *I. anguloclavatus*: Samples 5; 7–18; 20–22; 25–27; 30. *I. clifdenensis*: Samples 1; 3; 6; 8; 13; 16–19; 21; 23–24; 28; 32. *I. megagemmatus*: Samples 10; 15; 25 (all belonging to the MECO interval).

Periporopollenites demarcates Stover 1973. **Supplementary Figure 6.10**

This fossil species has been widely recorded both in Argentina (36, 50, 105, 119–120) and in Australia (e.g. Hill, 121–124) during warm periods from Maastrichtian to early Miocene.

Botanical affinity: The fossil pollen resembles those of *Trimenia papuana* Ridley and *T. weinmanniifolia* Seeman (Trimeniaceae) by having small pollen grains, spheroidal, with approximately 20 apertures, exine slightly punctate (125).

Geographical distribution of living representatives: Extant Trimeniaceae species are distributed in eastern Australia and an island chain stretching from Celebes to the Moluccas, New Guinea, New Caledonia, Fiji, Samoa, and the Marquesas. The family prefers a tropical climate moderated by the oceanic influence (126).

Distribution in Río Turbio Fm: Samples 7; 10; 13; 16–19; 25 (most of them belonging to the MECO interval).

Perisyncolporites pokornyi Germeraad, Hopping & Muller, 1968. **Figure 8.8**

*P. pokornyi*is a typical species recognized in fossil assemblages from South America and the Caribbean during warm periods from middle Eocene to Pleistocene (57, 75, 76–77, 127–136).

Botanical affinity: The pollen fossil analyzed resembles species of living Malpighiaceae, especially those of the Banisteroid group (137). Fossil forms are similar to some species of the Tetrapterid clade within the Banisteroid, currently present in Northern and central Argentina. They share the circular to subcircular outline, the thin colpoids, and a similar number of pores (138).

Geographical distribution of living representatives: The center of diversity of Malpighiaceae is tropical South America (139). *Gallardoa fischeri* Hicken (clade Cordobioide also included within the Banisteroide) represents the southernmost penetration of the family in the world,

reaching 41º S. That species differs from the specimens analyzed by showing verrucate sculpture (138).

Distribution in Río Turbio Fm: Samples 3 and 21.

Monocots

Araceae sp. 1. **Supplementary Figure 6.11**

Description: pollen grain subspherical to prolate, inaperturate, exine with 20-24 longitudinal ribs, sometimes bifurcated, that converge at the poles. Dimensions: equatorial diameter: 22-24 µm; polar diameter: 28-30 µm; ribs: 1 µm width; exine: 1 µm thick (3 specimens measured, one counted in this study).

Remarks: the RTF material presents most of the diagnostic features proposed by Hesse & Zetter (140) for the genus *Spathiphyllum*: Sphericity degree and thin and bifurcated ribs. The specimens studied not have more than 15 ribs per side.

Geographical distribution of living representatives: *Spathiphyllum* includes 50 species that exhibit a tropical distribution: 47 species are restricted to the Neotropics and the three remaining to the Philippines and Indonesia (141).

Distribution in Río Turbio Fm: Samples 11, 14, 16 and 18 (all belonging to the MECO interval)

Arecipites minutiscabratus? (McIntyre 1968) Milne 1988; *Arecipites regio* (Van der Hammen and Garcia, 1966) Jaramillo and Dilcher, 2001; *Psilamonocolpites medius* Van der Hammen 1954.

Botanical affinity: This morphotypes are comparable to those of family Arecaceae by having tectate exine and monosulcate aperture (142).

Geographical distribution of living representatives: Mainly tropical regions of the world (Africa, India, China, Taiwan, Vietnam, Indonesia, Sumatra, Thailand, Filipinas, Brazil, Central America and Caribbean Islands, Colombia, Bolivia, Perú, Argentina). In South America only *Trithrinax campestris* (Burmeist.) Drude & Griseb grows to the south of subtropical areas, reaching 32ºS (93).
Distribution in Río Turbio Fm: Sample 5.

Arecipites minutiscabratus? (McIntyre 1968) Milne 1988. **Not illustrated.**
Previous fossil records are from Argentina (39, 105, 143) and Australia (144–145) during warm periods from Maastrichtian to late Miocene.
Remarks: The tectate exine is clearly noted in intermediate focus in *Arecipites* (Fig. 6.11). The extended sulcus with pointed apices, as long as the long axis, has high systematic value (142, 146), it broadly link these morphotypes with some living subfamilies in the palm family as Coryphoideae, Calamoideae and Arecoideae.
Distribution in Río Turbio Fm: Samples 6; 13; 20–21(most of them belonging to the MECO interval).

Arecipites regio (Van der Hammen and Garcia, 1966) Jaramillo and Dilcher, 2001. **Supplementary Figure 6.12**
Fossil species found in late Paleocene to late Eocene of Colombia (52–53).
Remarks: This morphotype is comparable to those of family Arecaceae by having tectate exine, monosulcate aperture and finely pitted surface (142, 147).
Distribution in Río Turbio Fm: Samples 4; 9; 14; 21; 25.

Psilamonocolpites medius Van der Hammen 1954. **Figure 8.12**

Previous fossil records are from northern South America (68 and references there in) during warm periods from late Cretaceous to Oligocene.

Remarks: This morphotype is comparable to those of family Arecaceae by having tectate exine and monosulcate aperture (148).

Distribution in Río Turbio Fm: Samples 2–4; 6; 8–21; 23–26; 28-32.

Supplementary Note 5: R scripts

```
library(vegan)
library(rioja)
library(iNEXT)

rt<-read.csv(file="sup_table_2.csv",sep=",",header=TRUE)##Abundance matrix in csv;
 the oldest sample is number 1.
t_rt<-t(rt) #transpose matrix
rev<-t_rt[rev(rownames(t_rt)),]##to use the matrix reversed and transposed.

##Cluster Analysis (for either continental or marine abundance data)
# check total abundance in each sample
apply(rev,1,sum)
# Turn percent cover to relative abundance by dividing each value by sample
rot<-decostand(rev,method= "total")
# check total abundance in each sample
apply(rot,1,sum)
data.asin<-asin(sqrt(rot)) #arcsine square roottransformation
data.dist.q<-vegdist(data.asin,method="bray")
data.clust.q<-chclust(data.dist.q,method="coniss")
plot(data.clust.q,which.plots=2,hang=-0.1,cex=0.6,horiz=TRUE,x.rev=TRUE)

##MNDS
data.nms<-metaMDS(rot,distance="bray")# metaMDS is the function that does NMS
 usingbray-curtisdistance.
s1<-scores(data.nms,display=c("sites"),choices=1)#Axis 1 scores, Q-mode
s2<-scores(data.nms,display=c("sites"),choices=2)#Axis 2 scores, Q-mode
v1<-scores(data.nms,display=c("species"),choices=1)#Axis 1 score, R-mode
v2<-scores(data.nms,display=c("species"),choices=2)#Axis 2 score, R-mode

plot(data.nms,type="n")

points(s1[1:7],s2[1:7],pch=24,col="black",bg="gold")#post_meco
points(s1[8:25],s2[8:25],pch=21,col="black",bg="firebrick") #meco
points(s1[26:32],s2[26:32],pch=22,col="black",bg="darkgreen") #pre_meco

leg.text<-c("Post-MECO", "MECO", "Pre-MECO")#makes the legend
legend("bottomright",leg.text,pch=c(24,21,22),col=c("gold","firebrick","darkgreen")
 )

#Evenness (Evar)
library(codyn)
col1<-community_structure(rt, metric="Evar", abundance.var=1)
```

```
#Within-Sample Diversity (coverage at 0.8)
est<-estimateD(rt, datatype="abundance", base="coverage",level=0.8, conf=0.95)

##Within-Sample Diversity (Chao1 Estimato)r##
chao1<-iNEXT(rt, q=0, datatype="abundance", conf=0.95, nboot=100)

##Among-sample diversity
library(vegan)
pre_meco=specaccum(t_rt[1:7,],method="random")
meco=specaccum(t_rt[8:25,],method="random")
post_meco= specaccum(t_rt[26:32,], method = "random")
plot(meco, col="darkorange")
plot(pre_meco, col="darkgreen",add=TRUE)
plot(post_meco, col="gold",add=TRUE)
```

Supplementary References

1-Riccardi, A. C. & Rolleri, E. O. Cordillera patagónica austral. *Simposio de Geología Regional Argentina* **2**, 1173–1306 (1980).

2-Furque, G. & Caballé, M. Estudio geológico y geomorfológico de la cuenca superior del Río Turbio, provincia de Santa Cruz. *Consejo Federal de Inversiones, Serie Investigaciones aplicadas, Colección Hidrología subterránea* **6**, 8–39 (1993).

3-Malumián, N. in *Geología y recursos naturales de Santa Cruz* (ed Haller M. J.) 237–245 (Relatorio XV Congreso Geológico Argentino, Buenos Aires, 2002).

4-Guerstein, G. R. & Daners, G. Distribución de Enneadocysta (Dinoflagellata) en el Paleógeno del Atlántico Sudoccidental: implicancias paleoceanográficas. *Ameghiniana* **47**, 461–478 (2010b).

5-Malumián, N., Panza, J. & Parisi, C. Yacimiento Rio Turbio: Instituto de Geologia y Recursos Minerales SEGEMAR (Argentina) Carta Geológica 5172-III, escala, 1:250.000 (2000).

6-Leanza, A. F. in *Geología Regional Argentina* (ed. Leanza, A. F.) 689–706 (Academia Nacional de Ciencias, Córdoba, 1972).

7-Arguijo, M. H. & Romero, E. J. Análisis Bioestratigráfico de Formaciones portadoras de Tafofloras Terciarias. *Actas VIII Congreso Geológico Argentino* **6**, 691–717 (1981).

8-Ramos, V. A. in *Geología y recursos naturales de Santa Cruz* (ed Haller M. J.) 365–387 (Relatorio XV Congreso Geológico Argentino, Buenos Aires, 2002).

9-Hünicken, M. Flora Terciaria de los Estratos de Río Turbio, Santa Cruz (Niveles plantíferos del arroyo Santa Flavio). *Rev. Fac. Cienc. Exact., Fis. y Nat. Univ. Córdoba, S. Cs. Nat.* **27**, 139–227 (1967).

10-Panti, C. Myrtaceae fossil leaves from the Río Turbio Formation (Middle Eocene), Santa Cruz Province, Argentina. *Historical Biology* **28**, 459–469 (2016).

11-Panti, C. Southern beech (Nothofagaceae) fossil leaves from the Río Turbio Formation (Eocene–? Oligocene), Santa Cruz Province, Argentina. *Revista del Museo Argentino de Ciencias Naturales nueva serie* **21**, 69–85 (2019).

12-Panti, C. Fossil leaves of subtropical lineages in the Eocene–? Oligocene of southern Patagonia. *Historical Biology* **32**, 291–306 (2020).

13-Archangelsky, S. On the genus Tomaxellia (Coniferae) from the Lower Cretaceous of Patagonia (Argentina) and its male and female cones. *Botanical Journal of the Linnean Society* **61**, 153–165 (1968).

14-Archangelsky, S. Estudio del paleomicroplancton de la Formación Río Turbio (Eoceno), Provincia de Santa Cruz. *Ameghiniana* **6**, 181–218 (1969).

15-Archangelsky, S. & Fasola, A. Algunos elementos del Paleomicroplancton del Terciario inferior de Patagonia (Argentina y Chile). *Revista del Museo de la Plata* **6**, 1–18 (1971).

16-Archangelsky, S. Esporas de la Formación Río Turbio (Eoceno) Provincia de Santa Cruz. *Revista del Museo de la Plata* **6**, 65–100 (1972).

17-Romero, E. J. *Polen fósil de Gimnospermas y Fagáceas de la Formación Río Turbio (Eoceno), Santa Cruz, Argentina* (FECIC, Buenos Aires, 1977).

18-Archangelsky, S. & Romero, E. J. Polen de gimnospermas (coníferas) del Cretácico superior y Paleoceno de Patagonia. *Ameghiniana* **11**, 217–236 (1974a).

19-Archangelsky, S. & Romero, E. J. Los registros más antiguos del polen de Nothofagus (Fagaceas) de Patagonia (Argentina y Chile). *Boletín de la Sociedad de Botánica de México* **33**, 13–30 (1974b).

20-González Estebenet, M. S., Guerstein, G. R. & Rodríguez Raising, M. E. Middle Eocene Dinoflagellate cysts from Santa Cruz Province, Argentina: biostratigraphy and paleoenvironment. *Review of Paleobotany and Palynology* **211**, 55–65 (2014a).

21-González Estebenet, M. S., Guerstein, G. R. & Casadío, S. Estudio bioestratigráfico y paleoambiental de la Formation Río Turbio (Eoceno medio a tardío) en el sudoeste de Patagonia (Argentina) basado en quistes de dinoflagelados. *Revista Brasilera de Paleontología* **18**, 429–42 (2015).

22-González Estebenet, M. S., Guerstein, G. R., Rodriguez Raising, M. E., Ponce, J. J. &Alperín, M. I. Dinoflagellate cyst zonation for the middle to upper Eocene in the Austral Basin, southwestern Atlantic Ocean: implications for regional and global correlation. *Geological Magazine* **154**, 1022–1036 (2016).

23- Cramwinckel, M. J., Woelders, L., Huurdeman, E. P., Peterse, F., Gallagher, S. J., Pross, J., Burgess, C. E., Reichart, G. J., Sluijs, A. & Bijl, P. K. Surface-circulation change in the southwest Pacific Ocean across the Middle Eocene Climatic Optimum: inferences from dinoflagellate cysts and biomarker paleothermometry. *Climate of the Past* **16**, 1667–1689 (2020).

24-Rodríguez Raising, M. E. 2010. *Estratigrafía secuencial de los depósitos marinos y continentales del Eoceno –Oligoceno temprano de la cuenca Austral, sudoeste de la provincia de Santa Cruz.* Ph.D. thesis, Universidad Nacional del Sur, Buenos Aires, Argentina. Published thesis.

25-Fosdick, J. C., Van der Leest, R. A., Bostelmann, J. E., Leonard, J. S., Ugalde, R., Oyarzún, J. L. & Griffin, M. Revised timing of Cenozoic Atlantic incursions and changing hinterland sediment sources during southern Patagonian orogenesis. *Lithosphere* **1**, 1–18 (2020). doi:10.2113/2020/8883099

26-Bijl, P. K., Sluijs A. & Brinkhuis H. A magneto-and chemostratigraphically calibrated dinoflagellate cyst zonation of the early Palaeogene South Pacific Ocean. *Earth-Science Reviews* **124**, 1–31 (2013).

27-Guerstein, G. R., González Estebenet, M. S., Alperin M. I., Casadío S. A. & Archangelsky S. Correlation and paleoenvironments of middle Paleogene marine beds based on dinoflagellate cysts in southwestern Patagonia, Argentina. *Journal of South American Earth Sciences* **52**, 166–178 (2014).

28-Amenábar, C. R., Montes, M., Nozal, F. & Santillana, S. Dinoflagellate cysts of the La Meseta Formation (middle to late Eocene), Antarctic Peninsula: implications for biostratigraphy, palaeoceanography and palaeoenvironment. *Geological Magazine* **157**, 351–366 (2019).

29-Brinkhuis, H., Sengers, S., Sluijs, A., Warnaar, J. & Williams, G. L. in *Proceedings of the Ocean Drilling Program. Scientific Results* (eds Exon, N. F., Kennett, J. P. & Malone, M. J.) 1–48 (2003).

30-Sluijs, A., Brinkhuis, H., Stickley, C. E., Warnaar, J., Williams, G. L. & Fuller, M. in *Proceedings of the Ocean Drilling Program, Scientific Results* (eds Exon, N. F., Kennett J. P. & Malone, M. J.) 1–42 (ODP, 2003).

31-Houben, A. J., Bijl, P. K., Pross, J., Bohaty, S. M., Passchier, S., Stickley, C. E., Röhl U., Sugisaki S., Tauxe L., Flierdt T., Olney M., Sangiorgi F., Sluijs A., Escutia C., Brinkhuis H., Expedition 318 Scientists. Reorganization of Southern Ocean plankton ecosystem at the onset of Antarctic glaciation. *Science* **340**, 341–344 (2013).

32-Stickley, C. E., Brinkhuis, H., Schellenberg, S. A., Sluijs, A., Röhl, U., Fuller, M., Grauert, M., Huber, M., Warnaar, J. & Williams, G. L. Timing and nature of the deepening of the Tasmanian Gateway. Paleoceanography **19**, 4027 (2004).

33-González Estebenet, M. S., Guerstein, G. R. &Alperin, M. I. Dinoflagellate cyst distribution during the Middle Eocene in the Drake Passage area: paleoceanographic implications. *Ameghiniana* **51**, 500–9 (2014b).

34-Bijl, P. K., Pross, J., Warnaar, J., Stickley, C. E., Huber, M., Guerstein, R., Houben, A. J. P., Sluijs, A., Visscher, H. & Brinkhuis, H. Environmental forcings of Paleogene Southern Ocean dinoflagellate biogeography. *Paleoceanography***26**, 1202 (2011).

35-Archangelsky, S. Palinología del Paleoceno de Chubut. I. Descripciones Sistemáticas. *Ameghiniana* **10**, 339–399 (1973).

36-Mautino, L. R. & Anzótegui, L. M. Palinología de la Formación Chiquimil (Mioceno superior) en Vallecito, provincia de Catamarca, Argentina. Parte I. Esporas: Especies nuevas. *Ameghiniana* **35**, 227–233 (1998).

37-Ruiz, L. C. & Quattrocchio, M. E. Estudio Palinológico de la Formación Pedro Luro (?Maastrichtiano-Paleoceno) en la Cuenca del Colorado, República Argentina. Parte 1: Esporas triletes, Laevigati, Murornati, Tricassati, Cingulati y Zonati. *Revista Española de Micropaleontología* **29**, 13–29 (1997a).

38-Barreda, V. D., Gutiérrez, P. R. & Limarino,C. O. Edad y paleoambiente de la "Serie del Yeso", Valle del Cura, provincia de San Juan: evidencias palinológicas. *Ameghiniana* **35**, 321–335 (1998).

39-Barreda, V. D., Cúneo, N. R., Wilf, P., Currano, E. D., Scasso, R. A. & Brinkhuis, H. Cretaceous/Paleogene Floral Turnover in Patagonia: Drop in Diversity, Low Extinction, and a *Classopollis* Spike. *PLoSOne* **7**, 52455; 10.1371/journal.pone.0052455 (2012).

40-Scafati, L., Melendi, D. L. & Volkheimer, W. A Danian subtropical lacustrine palynobiota from South America (Bororó Formation, San Jorge Basin, Patagonia - Argentina). *Geological Acta* **7**, 35–61 (2009).

41-de Lima, M. R. & Boltenhagen, E. Estudo comparativo da evolução das microfloras Afro-Sul-Americanas II-O Cretáceo Superior. *Anais II Congresso Latino-Americano Paleontologia, Porto Alegre*, 373–383 (1981).

42-de Lima, M. R. Palinologia da Formaçao Codo Na Regiao de Codo, Maranhao. *Instituto de Geociências, Universidade de São Paulo* **13**, 43–134 (1982).

43-Garcia, M. J., Bistrichi, C. A., Saad, A. R., Campanha, V. A., & Oliveira, P. (2008). Stratigraphy and palaeoenvironments of the Tanque Basin, southeastern Brazil. *RevistaBrasileira de Paleontologia* **11**, 147–168.

44-Alroy, J., Marshall, C., & Miller, A. *Paleobiology database. NCEAS.* http://fossilworks.org/(2004).

45-Carrillo-Berumen, R., Quattrocchio, M. E. & Helenes, J. Paleogene continental Palynomorphs of the formations Chorrillo Chico and Agua Fresca, Punta Prat, Magallanes, Chile. *Andean Geology* **40**, 539–560 (2013).

46-Rouse, G. E. The application of a new nomenclatural approach to Upper Cretaceous plant microfossils from western Canada. *Canadian Journal of Botany* **35**, 349–375(1957).

47-Tryon, A. F. & Lugardon, B. *Spores of Pteridophyta* (Springer-Verlag, New York, 1991).

48- Morbelli, M. A. Morfología de las esporas de Pteridophyta presentes en la región fuego-patagónica. República Argentina. *Opera Lilloana* **28**, 1–138 (1980).

49-Moran, R. C. *American Genera of Ferns and Lycophytes, a guide for students. Version 2.1.* 618 pp. (The New York Botanical Garden, 2019).

50-Melendi, D. L., Scafati, L. H. & Volkheime W. Palynostratigraphy of the Paleogene Huitrera Formation in N-W Patagonia, Argentina. *Neues Jahrbuch für Geol. und. Pal., Abhandlungen* **228**, 205–273 (2003).

51-Clyde, W. C., Wilf, P., Slingerland, A. R. L., Barnum, T., Bijl, P. K., Bralower, T. J., Brinkhuis, H., Comer, E. E., Huber, B. T., Ibañez-Mejia Jicha, M. B. R., Krause, J. M., Schueth, J. D., Singer, B. S., Raigemborn, M. S., Schmitz, M. D., Sluijs, A. & Zamaloa, M. del C. New age constraints for the Salamanca Formation and lower Río Chico Group in the western San Jorge Basin, Patagonia, Argentina: Implications for Cretaceous-Paleogene extinction recovery and land mammal age correlations. *Geological Society America Bulletin* **126**, 289–306 (2014).

52-González-Guzmán, A. E. *A palynological study on the upper Los Cuervos and Mirador formations* (ed. Brill, E. J.) 68 pp. (Brill Archive, Leiden, Netherlands, 1967).

53-Jaramillo, C. A. & Dilcher, D. L. Middle Paleogene palynology of Central Colombia, South America: A study of pollen and spores from tropical latitudes. *Palaeontographica Abteilung B* **258**, 87–213 (2001).

54-Dettmann, M. E. Upper Mesozoic microfloras from south-eastern Australia. *Proceedings of the Royal Society of Victoria* **77**, 1–148 (1963).

55-Archangelsky, S., Baldoni, A., Gamerro, J. C. & Seiler, J. Palinología estratigráfica del Cretácico de Argentina austral. II. Descripcionessistemáticas. *Ameghiniana* **20**, 199–226 (1983).

56-Duarte, S. G., Mitsuru, A., & Wanderley, M. D. Morphometric study of fossil and extant spores of the Family Anemiaceae from the Lower Cretaceous to the Quaternary. *Revista do Instituto Geológico, São Paulo* **35**, 57–70 (2014).

57-Regali, M. S. P., Uesugui, N. & Santos, A. S. Palynology of the Mesozoic-Cenozoic sediments of Brazil. *Boletim Técnico da Petrobras (Rio de Janeiro)* **17**, 177–301 (1974).

58-Hoorn, C. Marine incursions and the influence of Andean tectonics on the Miocene depositional history of northwestern Amazonia: results of a palynostratigraphic study. *Palaeogeography, Palaeoclimatology, Palaeoecology* **105**, 267–309 (1993).

59-Hoorn, C. Palynology of the Pleistocene glacial/interglacial cycles of the Amazon Fan (Holes 940A, 944A, and 946A) in *Proceedings-ocean drilling program scientific results* (eds. Flood, R. D., Piper, D .J. W., Klaus, A., & Peterson, L. C.) 397–410 (National Science Foundation, 1997).

60-de Fátima Rossetti, D. Late Cenozoic sedimentary evolution in northeastern Pará, Brazil, within the context of sea level changes. *Journal of South American Earth Sciences* **14**, 77–89 (2001).

61-Hekel, H. Pollen and spore assemblages from Queensland Tertiary sediments. *Geological Survey of Queensland, Palaeontological paper* **30**, 34 (1972).

62-Macphail, M. K. &Truswell, E. M. Palynostratigraphy of the central west Murray Basin. *BMR Journal of Australian Geology and Geophysics* **11**, 3 (1989).

63-Macphail, M. K. Palynostratigraphy of the Murray Basin, inland southeastern Australia. *Palynology* **23**, 197–240 (1999).

64-Rozefelds, A. C., Dettmann, M. E., Clifford, H. T. & Carpenter, R. J. *Lygodium* (Schizaeaceae) in southern high latitudes during the Cenozoic—A new species and new insights into character evolution in the genus. *Review of Palaeobotany and Palynology* **247**, 40–52 (2017).

65-Tryon, R. & Tryon, A. F. Additional taxonomic and nomenclatural notes on ferns. *Rhodora* **84**, 125–130 (1982).

66-Wikström, N., Kenrick, P. & Vogel, J. C. Schizaeaceae: a phylogenetic approach. *Review of Palaeobotany and Palynology* **119**, 35–50 (2002).

67-Garrison Hanks, J. A Monographic study of Lygodium Swartz (Pteridophyta: Lygodiaceae) (The New York University, New York, 1998).

68-White, J. M. *Palynodata Datafile: 2006 version.* Canada. http://paleobotany.ru/ (2006).

69-Filatoff, J. Jurassic Palynology of the Perth Basin, Western Australia. *Palaeontographica, Abteilung B* **154**, 1–113 (1975).

70-Baldoni, A. M. & Archangelsky, S. Palinología de la Formación Springhill (Cretácico Inferior), subsuelo de Argentina y Chile austral. *Revista Española de Micropaleontología* **15**, 47–101 (1983).

71-Sajjadi, F. & Playford, G. Systematic and stratigraphic palynology of Late Jurassic – earliest Cretaceous strata of the Eromanga basin, Queensland, Australia: Part Two. *Palaeontographica* B **261**, 99–165 (2002).

72-Cranwell, L. M. & Srivastava, S. K. An early Cretaceous (Hauterivian) spore pollen assemblage from Southern Chile. *Palynology* **33**, 241–280 (2009).

73-Garcia, G. G., Garcia, A. J. V. & Henriques, M. H. P. Palynology of the Morro do Chaves Formation (Lower Cretaceous), Sergipe Alagoas Basin, NE Brazil: Paleoenvironmental implications for the early history of the South Atlantic. *Cretaceous Research* **90**, 7–20 (2018).

74-Harris, T. M. The Yorkshire Jurassic Flora I, Thallophyta—Pteridophyta. British Museum (Natural History), London (1961).

75-Dueñas-Jimenez, H. Geología y palinología de la Formación Ciénaga de oro, región Caribe Colombiana. *República de Colombia, Ministerio de Minas y Energía Instituto Nacional de Investigaciones Geológico-Mineras*, **18** (1986).

76-Hoorn, C., Raghubanshi, A.S. & Singh, J.S. 1994. Fluvial palaeoenvironments in the intracratonic Amazonas Basin (Early Miocene - Early Middle Miocene, Colombia). *Palaeogeography, Palaeoclimatology, Palaeoecology* **109**, 1–54.

77-Rull, V. Sequence analysis of western Venezuelan Cretaceous to Eocene sediments using palynology: chronopaleoenvironmental and paleovegetational approaches. *Palynology* **21**, 79–90 (1997).

78-Rull, V. A quantitative palynological record from the early Miocene of western Venezuela, with emphasis on mangroves. *Palynology* **25**, 109–126 (2001).

79-Martin, H. A. The Tertiary stratigraphic palynology of the Murray Basin in New South Wales. 1: The Hay-Balranald-Wakool District. *Proceedings of the Royal Society of New South Wales* **110**, 41–47 (1977a).

80-Christophel, D. C., Harris, W. K. & Syber, A. K. The Eocene flora of the Anglesea locality, Victoria. *Alcheringa* **11**, 303–323 (1987).

81-Zamaloa, M. del C. & Romero, E. J. Some spores and pollen from the Cullen Formation (upper Eocene to middle Oligocene), Tierra del Fuego, Argentina. *Palynology* **14**, 123–133 (1990).

82-Mohr, B. A., & Lazarus, D. B. Paleobiogeographic distribution of *Kuylisporites* and its possible relationship to the extant fern genus *Cnemidaria* (Cyatheaceae). *Annals of the Missouri Botanical Garden* **81**, 758–767 (1994).

83-Archangelsky, S. Palinología del Paleoceno de Chubut. II. Diagramas polínicos. *Ameghiniana* **13**, 43–55 (1976).

84-Papú, O. H. Estudio palinológico de la Formación Paso del Sapo (Cretácico Superior), Valle Medio del Río Chubut. Granos de polen, consideraciones estadísticos, paleoecológicas y paleoambientales. *Ameghiniana* **25**, 193–202 (1989).

85-Ruiz, L. C. & Quattrocchio, M. E. *Srivastavapollenites exoticus* nov. Gen et sp. de la Formación Pedro Luro (Paleoceno), Cuenca del Colorado, Argentina. *Ameghiniana* **30**, 311–315 (1993).

86-Ruiz, L. C. & Quattrocchio, M. Stratigraphic palynology of the Pedro Luro formation (?Maastrichtian-Paleocene), Colorado Basin, Argentina. *Bulletin des Centres de recherches exploration-production Elf-Aquitaine. Mémoire* **16**, 361–371 (1996).

87-Ruiz, L.C. & Quattrocchio, M.E. Estudio palinológico de la Formación Pedro Luro (?Maastrichtiano-Paleoceno), en la Cuenca del Colorado, República Argentina. Parte 2: Turma Saccites, Plicates, Poroses e Incertae Sedis. *Revista Española de Micropaleontología* **29**, 115–137(1997b).

88-Malécot, V. & Lobreau-Callen, D. A survey of species assigned to the fossil pollen genus *Anacolosidites*. *Grana* **44**, 314–336 (2005)

89-Mildenhall, D.C. Palynomorphs from Miocene – Pliocene sediments, Grey Valley (K31-Metric), South Island, New Zealand. *New Zealand Geological Survey Report PAL* **24**, 1–17 (1978).

90-Raine, J. I. Outline of a palynological zonation of Cretaceous to Paleogene terrestrial sediments in west coast region, South Island, New Zealand. *New Zealand Geol. Surv. Rep.* **109**, 1–82 (1984).

91-Raine, J. I. & Wilson, G. J. Palynology of the Mt Somers (South Island, New Zealand) early Cenozoic sequence (note). *New Zealand journal of geology and geophysics* **31**, 385–390 (1988).

92-Pocknall, D. T. Palynological evidence for the early to middle Eocene vegetation and climate history of New Zealand. *Review of palaeobotany and palynology* **65**, 57–69 (1990).

93-Zuloaga, F., Morrone, O. & Belgrano, M. *Flora del Cono Sur. Catálogo de las Plantas Vasculares. Instituto de Botánica Darwinion, Buenos Aires.* www2. darwin. edu. ar/Proyectos/FloraArgentina/FA. asp. (2009).

94-Mildenhall, D. C. & Pocknall, D. T. *Miocene–Pleistocene spores and pollen from Central Otago, South Island, New Zealand. New Zealand Geological Survey Palaeontological Bulletin no. 59*, 128 pp. (1989).

95-Acevedo-Rodríguez, P., Wurdack, K. J., Ferrucci, M. S., Johnson, G., Dias, P., Coelho, R. G., Somner, G. V., Steinmann,V. W., Zimmer, E. A. & Strong, M. T. Generic relationships and classification of tribe Paullinieae (Sapindaceae) with a new concept of supertribePaulliniodae. *Systematic Botany* **42**, 96–114 (2017).

96-Barreda, V. D. Bioestratigraffa de polen y esporas de la Formación Chenque, Oligoceno Tardío?-Mioceno de las provincias de Chubut y Santa Cruz, Patagonia, Argentina. *Ameghiniana* **33**, 35–56(1996).

97-Barreda, V. D. Palynomorph assemblage of the Chenque Formation, Late Oligocene?-Miocene from Golfo San Jorge basin, Patagonia, Argentina. Part 3. Polycolpate and tricolporate pollen. *Ameghiniana* **34**, 131–144 (1997).

98-Barreda, V. D. & Caccavari, M. Mimosoideae (Leguminosae) occurrences in the early Miocene of Patagonia (Argentina). *Palaeogeography, Palaeoclimatology, Palaeoecology* **94**, 243–252 (1992).

99-Barreda, V. & Palamarczuk, S. Palinoestratigrafía de depósitos del Oligoceno tardío-Mioceno en el área sur del Golfo San Jorge, provincia de Santa Cruz, Argentina. *Ameghiniana* **37**, 103–117(2000).

100-Palamarczuk, S. & Barreda, V. D. Late Paleogene-Early Neogene palynology, Aries x-1 well, Argentine continental shelf, Tierra del Fuego, Argentina. *Ameghiniana* **37**, 221–234 (2000).

101-Herngreen, G. F. W. Microfloral relationships between Africa and South America in *Middle and Upper Cretaceous time. International Palynological Conference, 4th Proceedings, Lucknow, 1976-1977*406–417 (Birbal Sahni Institute of Palaeobotany, Lucknow, India, 1981).

102-Beurlen, G. &, M. S. P. O Cretáceo da plataforma continental do Maranhão e Pará, Brasil: bioestratigrafia e evolucãopaleoambiental. *Bol. Geoc. PETROBRAS* **1**, 135–155 (1987).

103-Viviers, M. C., & Regali, M. S. P. Estudo paleoambiental preliminar do Cretáceo da Bacia Potiguar. *Revista Brasileira de Geociências* **17**, 123–130 (2018).

104-Bellonzi, T. K., Dutra, F. V., Souza, C. N. D., Rezende, A. A. & Gasparino, E. C. Pollen types of Sapindaceae from Brazilian forest fragments: apertural variation. *Acta Botanica Brasilica* **34**, 327–341(2020).

105-Romero, J. E. & Castro, M. T. Material fúngico y granos de polen de angiospermas de la Formación Río Turbio (Eoceno), provincia de Santa Cruz, República Argentina. *Ameghiniana* **23**, 101–118 (1986).

106-Anzótegui, L. M. & Acevedo, T. L. Revisión de*Ilexpollenitesthiergartyuna* nueva especieen el PliocenoSuperior (FormaciónItuzaingó) de Corrientes, Argentina. *Actas de la VI Congreso Argentino de Paleontología y Bioestratigrafía, Trelew*, 15–21 (1995).

107-Povilauskas, L. Palinología de angiospermas de la Formación Monte Chico (Cretácico Superior) de la Provincia de Santa Cruz, Argentina. *Revista brasileira de paleontología* **16**, 115–126 (2013).

108-Stover, L. E. & Partridge, A. D. Tertiary and Late Cretaceous spores and pollen from the Gippsland Basin, southeastern Australia. *Proceedings of the Royal Society of Victoria* **85**, 237–286 (1973).

109-Stover, L. E. & Partridge, A. D. Eocene spore-pollen from the Werillup Formation, Western Australia. *Palynology* **6**, 69–96 (1982).

110-Martin, H. A. The history of *Ilex* (Aquifoliaceae) with special reference to Australia: evidence from pollen. *Australian journal of botany* **25**, 655–673 (1977b).

111-Martin, H. A. Tertiary stratigraphic palynology and palaeoclimate of the inland river systems in New South Wales. *The Cainozoic in Australia: a re-appraisal of the evidence*. *Special Publication* **18**, 181–194 (1991).

112-Tulip, J. R., Taylor, G. & Truswell, E. M. Palynology of Tertiary Lake Bunyan, Cooma, New South Wales. *Bureau of Mineral Resources journal of Australian geology and geophysics* **7**, 255–268 (1982).

113-Truswell, E. M. & Owen, J. A. Eocene pollen from Bungonia, New South Wales. *Memoir of the Association of Australasian Paleontologists* **5**, 259–284 (1988).

114-Alley, N.F. & Benbow, M. C. Late Eocene palynofloras from the Pidinga Formation SADME Ooldea Range 6, eastern Eucla Basin. *Geological Survey of South Australia Quarterly Geological Notes* **111**, 2–12 (1989).

115-Dettmann, M .E. & Jarzen, D. M. The Antarctic/Australian rift valley: Late cretaceous cradle of northeastern Australasian relicts? *Review of Palaeobotany and Palynology* **65**, 131–144 (1990).

116-Pocknall, D. T. Palynostratigraphy of the TeKuiti Group (late Eocene-Oligocene), Waikato Basin, New Zealand. *New Zealand journal of geology and geophysics* **34**, 407–417 (1991).

117-Heywood, V. H. *The Flowering Plants of the World*. Oxford University Press, Oxford, pp. 335 (1978).

118-Macphail, M. K., Alley, N. F., Truswell, E. M. & Sluiter, I. R. K. Early Tertiary vegetation: evidence from spores and pollen in *History of the Australian vegetation: Cretaceous to Recent* (ed. Hill, R. S.) 189–261 (University of Adelaide Press, 1994).

119-Baez, A. M., Zamaloa, M. del C. & Romero, E. J. Nuevos hallazgos de microfloras y anuros Paleógenos en el Noroeste de Patagonia: implicancias Paleoambientales y Paleobiogeográficas. *Ameghiniana* **27**, 83–94 (1990).

120-Romero, J.E. & Zamaloa, M. del C. Polen de angiospermas de la Formación Río Turbio (Eoceno), provincia de Santa Cruz, Argentina. *Ameghiniana* **22**, 43–51 (1985).

121-Hill, R. S. Tertiary Nothofagus macrofossils from Cethana, Tasmania. *Alcheringa* **8**, 81–86 (1984).

122-Hill, R. S. Leaves of *Eucryphia* (Eucryphiaceae) from tertiary sediments in south-eastern Australia. *Australian Systematic Botany* **4**, 481–497 (1991).

123-Carpenter, R. J., Jordan, G. J. & Hill, R. S. A Toothed Lauraceae Leaf from the Early Eocene of Tasmania, Australia. *International Journal of Plant Sciences* **168**, 1191–1198 (2007).

124-Taylor, G., Truswell, E. M., McQueen, K. G. & Brown, M. C. Early Tertiary palaeogeography, landform evolution, and palaeoclimates of the Southern Monaro, N.S.W., Australia. *Palaeogeography, Palaeoclimatology, Palaeoecology* **78**, 109–134 (1990).

125-Sampson, F. B. & Endress, P. K. Pollen morphology in the Trimeniaceae. *Grana* **23**, 129–137 (1984).

126-Yamada, T., Nishida, H., Umebayashi, M., Uemura, K., & Kato, M. Oldest record of Trimeniaceae from the Early Cretaceous of northern Japan. *BMC Evolutionary Biology* **8**, 135 (2008).

127-Porta, J. D. *Colombie (deuxiemepartie). Tertiare et Quaternaire. LexiqueStratigraphique International, AmeriqueLatine, V (4b).*689 pp. (Centre National de la Recherche Scientifique, Paris, 1974)

128-Doubinger, J. Spore–pollen assemblages from the Tertiary of Colombia. *Acts, 97 Contres Nat.'l des Soc. Savantes (Nantes)* **4**, 9–18 (1976).

129-Dueñas-Jimenez, H. Sequence analysis of Western Venezuelan Cretaceous to Eocene sediments using palynology: Chrono-paleoenvironmental and paleovegetational approaches: Discussion and reply. Discussion. *Palynology* **23**, 31–33 (1999).

130-de Lima, M. R., & Salard-Cheboldaeff, M. Palynologie des bassins de Gandarela et Fonseca (Eocene de l'etat de Minas Gerais, Bresil). *Boletim IG-USP, Série Científica* **12**, 33–53(1981).

131-de Lima, M. R., & Dino, R. Palinologia de amostras da bacia de Bonfim, Terciário do Estado de São Paulo, Brasil. *Boletim IG-USP, Série Científica* **15**, 1–11(1984).

132-Escobar, L. E. Estudio palinológico de la Formación Amaga. *Boletín de Cienciasde la Tierra*, *Universidad Nacional de Colombia* **7**, 117–129 (1984).

133-de Lima, M. R., Salard-Cheboldaeff, M. & Suguio, K. Étudepalynologique de la FormationTremembé, Tertiairie du Bassin de Taubaté, (Etat de São Paulo, Brésil), d'après les echantillons du sondage no. 42 du CNP in *Coletânea de Trabalhos Paleontológicos, Brasília, Departamento Nacional da Produção Mineral. SérieGeologia 27, SeçãoPaleontologia e Estratigrafia* (eds. Campos, D.A., Ferreira, C.S., Brito I.M., Viana, C.F.) 379–393 (1985).

134-Fasola, A. & Paredes De Ramos, I. Late Cretaceous palynological assemblages from El Furrial area wells. *Revista Técnica Intevec* **11**, 3–3 (1991).

135-Colmenares, O. A. & Teran, L. A biostratigraphic study of Paleogene sequences insouthwestern Venezuela. *Palynology* **17**, 67–89 (1993).

136-Jaramillo, C.A. & Dilcher, D. L. Microfloral diversity patterns of the latePaleocene–Eocene interval in Colombia, northern South America. *Geology* **28**, 815–818 (2000).

137-Davis, C. C. & Anderson, W. R. A complete generic phylogeny of Malpighiaceae inferred from nucleotide sequence data and morphology. *American Journal of Botany* **97**, 2031–2048 (2010).

138-Fernández, D. A., Panti, C., Palazzesi, L. & Barreda, V. D. La presencia de una familia neotropical (Malpighiaceae) en el extremos más austral de Sudamérica durante el Eoceno. *Revista brasileira de paleontologia* **15**, 386–391 (2012).

139- Anderson, W. R. Malpighiaceae (Malpighia family) in *Flowering plants of the Neotropics* (eds. Smith, N., Mori, S.A., Henderson, A., Stevenson, D.W., Heald, S.V.). 229–232 (Princeton Univ Press. 2004).

140-Hesse, M. & Zetter, R. The fossil pollen record of Araceae. *Plant Systematics and Evolution* **263**, 93–115 (2007).

141-Cardona, F. Synopsis of the genus Spathiphyllum (Araceae) in Colombia. *Annals of the Missouri Botanical Garden* **91**, 448–456 (2004).

142-Harley, M. M. A summary of fossil records for Arecaceae. *Botanical Journal of the linnean Society* **151**, 39–67 (2006).

143-Herbst, R., Anzótegui, L. M., Esteban, G., Mautino, L. R., Morton, S. & Nasif, N. (2000). Síntesis paleontológica del Mioceno de los valles Calchaquíes, noroeste argentino in *El Neógeno de Argentina: INSUGEO, Serie Correlación Geológica, vol. 14* (eds. Herbst, R., Aceñolaza, F. G.) 263–288 (Ediciones Magna Publicaciones. 2000).

144-Milne, L. A., 1988. Palynology of a late Eocene lignitic sequence from the western margin of the Eucla Basin, Western Australia. *Association of Australasian Palalaeontologists Memoir* **5**, 285–310.

145-Martin, H. A. Late Cretaceous-Cainozoic palynology of the poonarunna no. 1 well, central Australia. *Transactions of the Royal Society of South Australia* **122**, 89–138 (1998).

146-Harley, M. M. & Baker, W. J. Pollen aperture morphology in Arecaceae: Application within phylogenetic analyses, and a summary of record of palm-like pollen the fossil. *Grana* **40**, 45–77 (2001).

147-Nichols, D. J., Ames, H. T. & Traverse, A. On Arecipites Wodehouse, *Monocolpopollenites* Thomson & Pflug, and the species *Monocolpopollenites tranquillus*. *Taxon* **22**, 241–256 (1973).

148-Jaramillo, C. & Rueda, M. *A Morphological Electronic Database of Cretaceous-Tertiary and Extant pollen and spores from Northern South America, v. 2020.*http://biogeodb.stri.si.edu/jaramillosdb/web/morphological/(2020).

149-Exon, N. F., Kennett, J. P., Malone, M. J., et al. Proceedings of the Ocean Drilling Program, Initial Reports Volume 189 (2001).

150-Williams, G. L., Fensome, R. A. & MacRae, R. A. The Lentin and Williams index of fossil dinoflagellates. American Association of Stratigraphic Palynologists Contributions Series **48** (2017).

Supplementary Figures

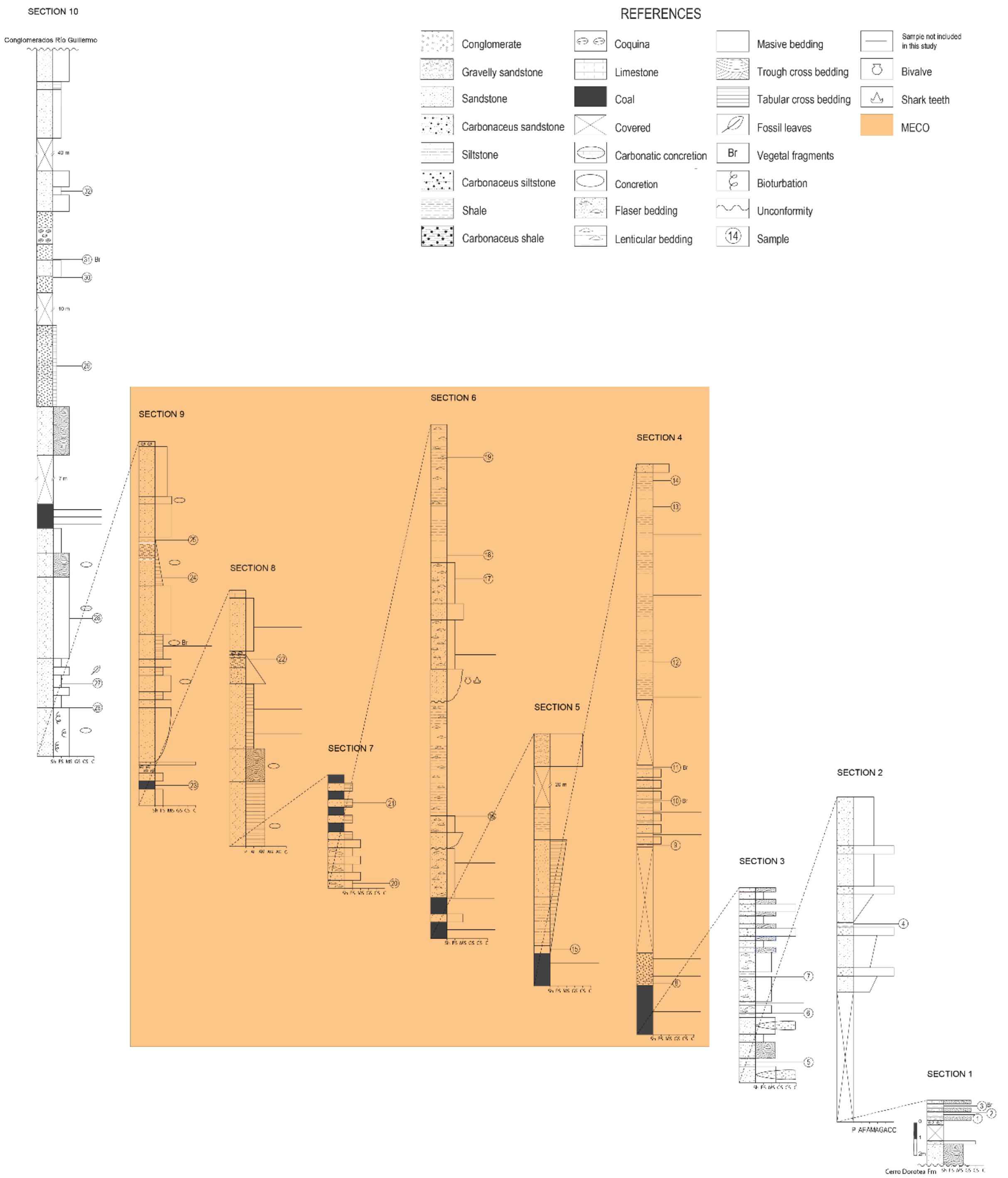

Supplementary Figure 1. Schematic correlation of the Río Turbio Formation showing sections and sample locations. Section 1 (51°33'24.40"S 72°21'5.90"W); Section 2 (51°31'57.60"S 72°19'13.30"W); Section 3 (51°34'18.96"S 72°20'37.14"W); Section 4 (51°32'29.40"S 72°19'40.70"W); Section 5 (51°32'29.40"S 72°19'40.70"W); Section 6

(51°32'29.30"S 72°19'40.40"W); Section 7 (51°30'39.20"S 72°15'38.40"W); Section 8 (51°30'37.00"S 72°15'33.00"W); Section 9 (51°29'24.10"S 72°14'43.15"W); Section 10 (51°26'0.30"S 72°13'41.30"W). Selected samples marked with numbers.

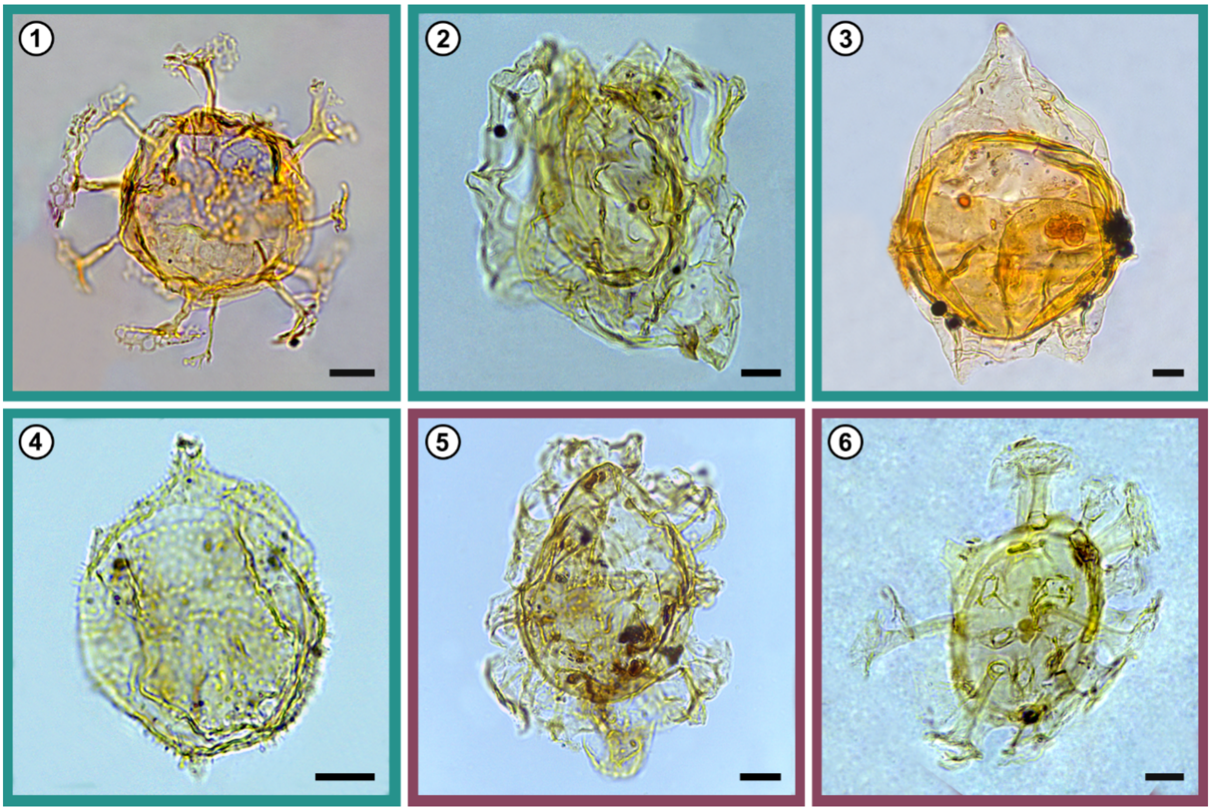

Supplementary Figure 2. Selected dinocysts species from the Eocene of southern South America. 1–4. Endemic elements (green square); 5–6. Cosmopolitan elements (purple square). 1. *Enneadocysta dictyostila,* sample 10 M41(4); 2. *Arachnodinium antarcticum,* sample 17 N38(1); 3. *Deflandrea antarctica,* sample 18Q38(3); 4. *Vozzhennikovia apertura,* sample 26 O44(4); 5. *Turbiospherafilosa,* sample 26 U40(1); 6. *Hystrichosphaeridium truswelliae,* sample 5F44(1). Scale bar is 10 µm. Taxonomic names are followed by the slide number and England Finder coordinates.

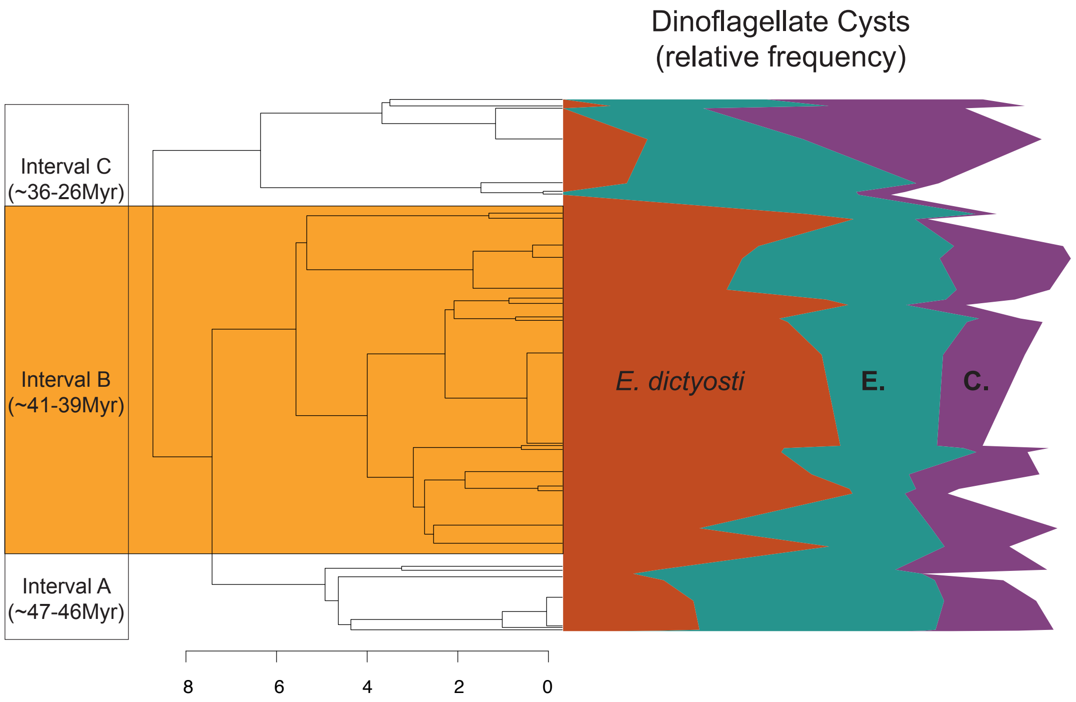

Supplementary Figure 3. Trends in frequency of our marine components (i.e. dinocysts). We identified three major biogeographic groups in southern Patagonia: *Enneadocysta dictyostila*, E=Endemics (e.g. *Vozzhennikovia apertura*, *Spinidinium macmurdoense*, *Deflandrea antarctica*, *Arachnodinium antarcticum*, *Enneadocysta brevistila*, *Impletosphaeridium clavus*), C=Cosmopolitan (e.g. *Turbiosphaera filosa*, *Thalassiphora pelagica*, *Spiniferites* spp. *Operculodinium* spp., *Hystrichosphaeridium truswelliae*). The response of these biogeographic groups from southern Patagonia to the MECO shows a very close similarity with that previously reported in Australia (6–7), and Antarctica (31–33) (see Supplementary Note 2 for further information).

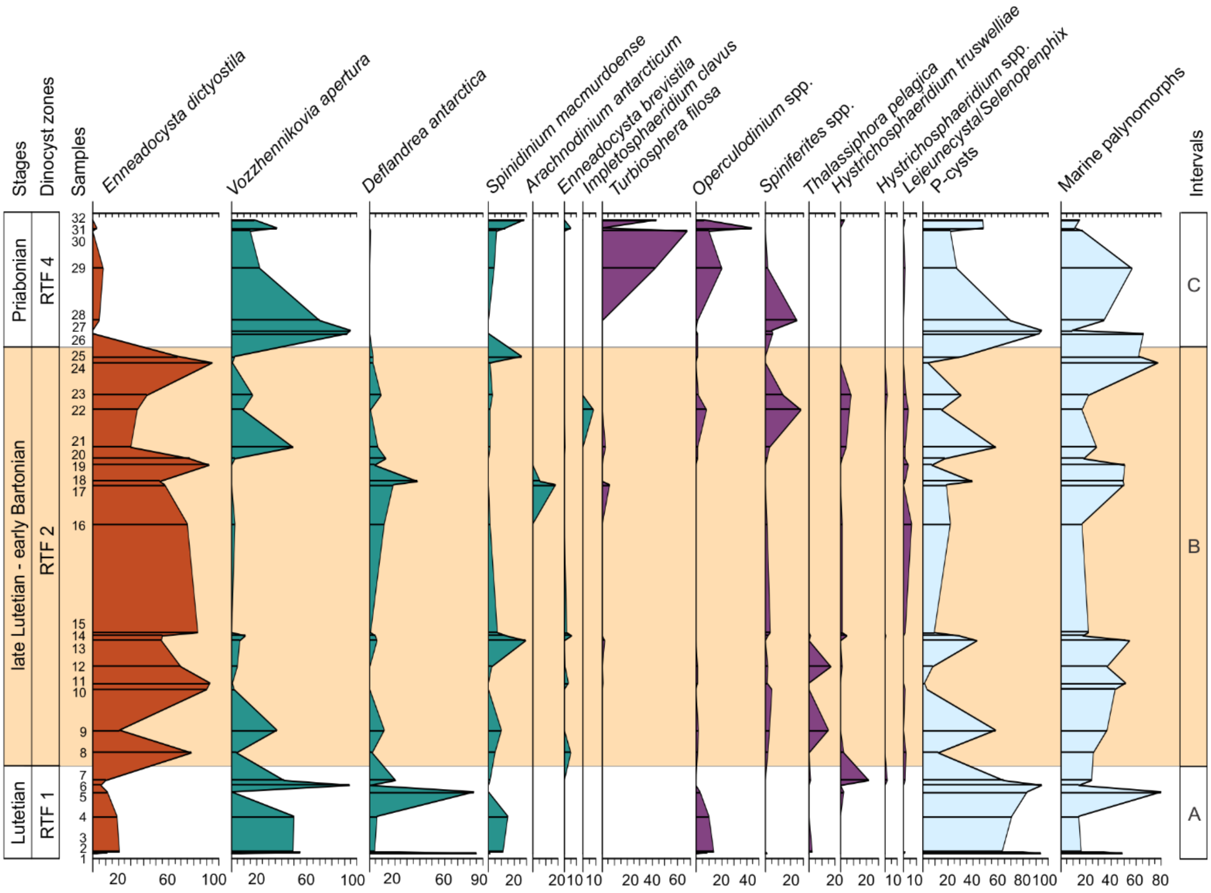

Supplementary Figure 4. Quantitative distribution of the dinocyst assemblages from the Río Turbio Formation. Distinct dinocyst biogeographic groups: Antarctic endemic taxa (*Enneadocysta dictyostila, Vozzhennikovia apertura*, *Spinidinium macmurdoense*, *Deflandrea antarctica*, *Arachnodinium antarcticum*, *Enneadocysta brevistila* and *Impletosphaeridium clavus*) and cosmopolitan (e.g. *Turbiosphaera filosa*, *Thalassiphora pelagica*, *Spiniferites* spp., *Operculodinium* spp., *Hystrichosphaeridium truswelliae*). The latitudinal distribution is based on Bijl et al. (26), Bijl et al. (34), Houben et al. (31) and Cramwinckel et al., (23). P-cysts: percentages of peridinioid cysts (P-cysts) over total of dinocysts. Marine palynomorphs: percentage of dinocysts over total of palynomorphs. The dinocyst zones follow González Estebenet et al. (22).

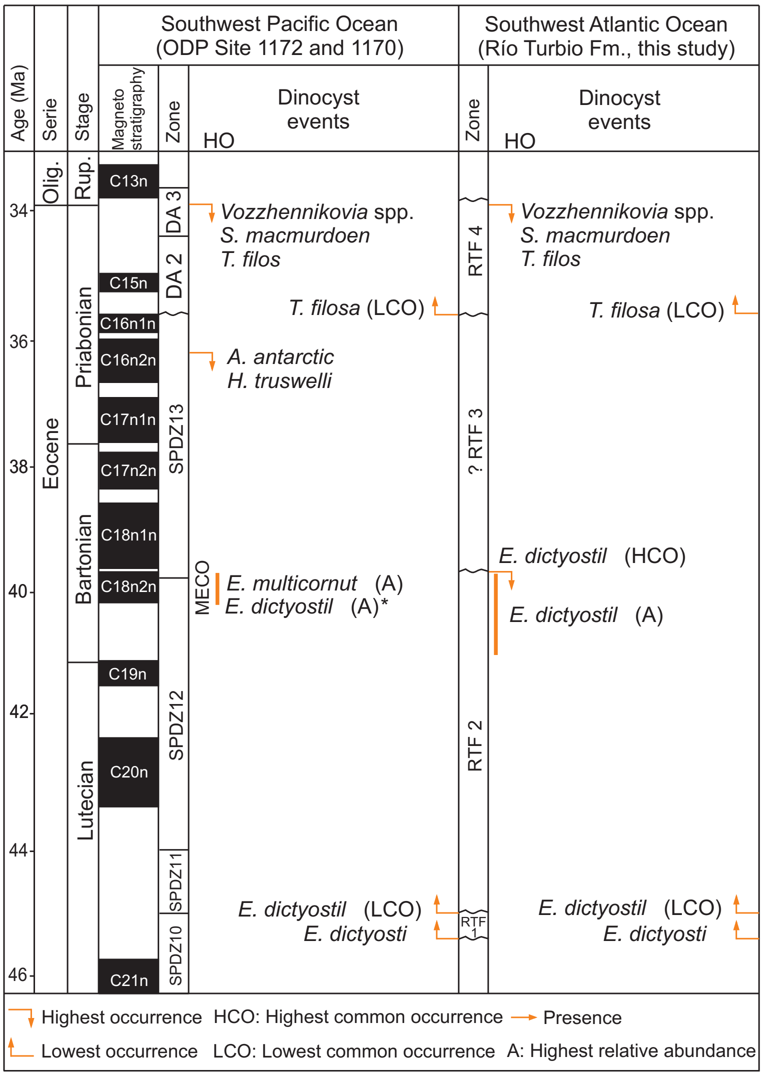

Supplementary Figure 5. Dinocyst events and zones recorded in the Río Turbio Formation (22; this study). Comparison with dinocyst events and zonation schemes from South Pacific Ocean (ODP Site 1172;26, 29, 30). The bioevent data marked by asterisk (*) come from ODP Site 1170 (149). DA: Dinocyst Association (30); SPDZ: South Pacific Dinocyst Zones (26). RTF: dinocyst zones of the upper member of the Río Turbio Formation (22).

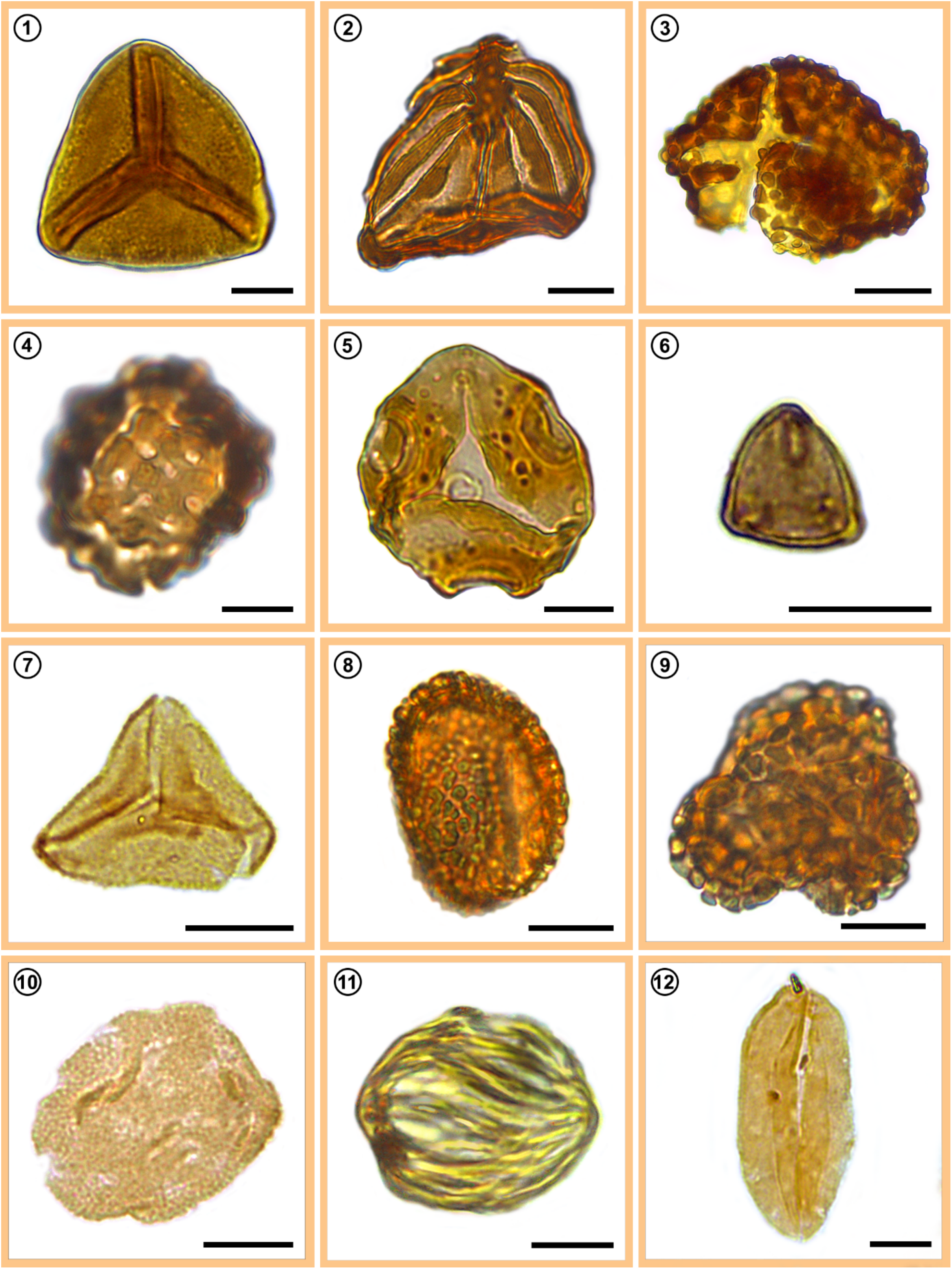

Supplementary Figure 6. Fossil forms with tropical and subtropical affinity from the RTF. 1. *Biretisporites crassilabratus,* sample 21 M8(3); 2. *Cicatricosisporites* sp. 4, sample 11 F55(4); 3. *Crassoretitriletes vanraadshooveni,* sample 12T12(2); 4. *Klukisporites scaberis,*

sample 25 O50(3); 5. *Kuylisporites waterbolkii,* sample 09 C17(1); 6.*Anacolosidites diffusa,* sample 30X53(3); 7. *Cupanieidites reticularis,* sample 14M20(2); 8. *Ilexpollenites clifdenensis,* sample 16 T28(1); 9. *I. megagemmatus,* sample 25 E32(4); 10. *Periporopollenites demarcatus*, sample 13 J45(2); 11. Araceae sp. 1., sample 5 P35(3); 12. *Arecipites regio*, sample 25 M37(1). Scale bar is 10 µm. Taxonomic names are followed by the slide number and England Finder coordinates.

Supplementary Data 1. Frequency of dinocysts identified in the Río Turbio Formation. Species taxonomy follows Williams et al. (150). Its latitudinal distribution is based on Bijl et al. (34), Bijl et al. (26) and Houben et al. (31) and Cramwinckel et al. (23).

Supplementary Data 2. Species list, botanical affinity, distribution and abundance data of the spore-pollen assemblages.
