# Supplementary material for: Impact of mid Eocene greenhouse warming on America’s southernmost floras
Source: Commun Biol. 2021 Feb 9;4:176. doi: 10.1038/s42003-021-01701-5 (PMC7873257; doi:10.1038/s42003-021-01701-5)
Supplement: Supplementary file 3 — Description of Supplementary Files [file 42003_2021_1701_MOESM3_ESM.pdf]

Description of Additional Supplementary Files

File name: Supplementary Data 1

Description: Supplementary Data 1. Species list, latitudinal distribution and abundance data of the dinocyst assemblages.

File name: Supplementary Data 2

Description: Species list, botanical affinity, distribution and abundance data of the spore-pollen assemblages. List of *Arabidopsis thaliana* accessions used in this study, along with their chlorophyll (Chl) contents under the control N conditions (6 mM N; $Chl_{control}$) and low N condition ($Chl_{low\ N}$). Data represent mean ± S.D. of five biological replicates.
