# Supplementary material for: Impact of mid Eocene greenhouse warming on America’s southernmost floras
Source: Commun Biol. 2021 Feb 9;4:176. doi: 10.1038/s42003-021-01701-5 (PMC7873257; doi:10.1038/s42003-021-01701-5)
Supplement: Supplementary file 6 — Reporting Summary [file 42003_2021_1701_MOESM6_ESM.pdf]

nature research

Corresponding author(s): Luis Palazzesi

Last updated by author(s): Dec 2, 2020

Reporting Summary

Nature Research wishes to improve the reproducibility of the work that we publish. This form provides structure for consistency and transparency in reporting. For further information on Nature Research policies, see our Editorial Policies and the Editorial Policy Checklist.

Statistics

For all statistical analyses, confirm that the following items are present in the figure legend, table legend, main text, or Methods section.

n/a	Confirmed	
☐	☒	The exact sample size (*n*) for each experimental group/condition, given as a discrete number and unit of measurement
☐	☒	A statement on whether measurements were taken from distinct samples or whether the same sample was measured repeatedly
☐	☒	The statistical test(s) used AND whether they are one- or two-sided<br>*Only common tests should be described solely by name; describe more complex techniques in the Methods section.*
☒	☐	A description of all covariates tested
☒	☐	A description of any assumptions or corrections, such as tests of normality and adjustment for multiple comparisons
☒	☐	A full description of the statistical parameters including central tendency (e.g. means) or other basic estimates (e.g. regression coefficient) AND variation (e.g. standard deviation) or associated estimates of uncertainty (e.g. confidence intervals)
☒	☐	For null hypothesis testing, the test statistic (e.g. F, t, r) with confidence intervals, effect sizes, degrees of freedom and P value noted<br>*Give P values as exact values whenever suitable.*
☒	☐	For Bayesian analysis, information on the choice of priors and Markov chain Monte Carlo settings
☒	☐	For hierarchical and complex designs, identification of the appropriate level for tests and full reporting of outcomes
☒	☐	Estimates of effect sizes (e.g. Cohen's d, Pearson's r), indicating how they were calculated

Our web collection on statistics for biologists contains articles on many of the points above.

Software and code

Policy information about availability of computer code

Data collection	As any palaeontological study, ours include the collection of data during fieldwork (clearly specified in the manuscript) with the use of any software.
Data analysis	We analyzed our data using the open source software R 3.6.1 (2019-07-05), with several packages, as specified in the manuscript.

For manuscripts utilizing custom algorithms or software that are central to the research but not yet described in published literature, software must be made available to editors and reviewers. We strongly encourage code deposition in a community repository (e.g. GitHub). See the Nature Research guidelines for submitting code & software for further information.

Data

Policy information about availability of data

All manuscripts must include a data availability statement. This statement should provide the following information, where applicable:

- Accession codes, unique identifiers, or web links for publicly available datasets
- A list of figures that have associated raw data
- A description of any restrictions on data availability

Accession codes of the housed slides containing fossil specimens are provided in the manuscript; the specimens are housed at the Museo "Padre Jesús Molina" under the catalogue numbers 21647–21699, prefixed MPM-PB.

Field-specific reporting

Please select the one below that is the best fit for your research. If you are not sure, read the appropriate sections before making your selection.

☐ Life sciences ☐ Behavioural & social sciences ☒ Ecological, evolutionary & environmental sciences

For a reference copy of the document with all sections, see nature.com/documents/nr-reporting-summary-flat.pdf

Ecological, evolutionary & environmental sciences study design

All studies must disclose on these points even when the disclosure is negative.

Study description	We quantified plant species richness in the southernmost regions of South America during the globally warm mid Eocene Epoch using palynological data (pollen and spores).
Research sample	The research sample includes fossil remains (plants), namely: spores, pollen grans and dinoflagellate cysts, recovered form Eocene rocks
Sampling strategy	We used rarefaction from relative abundance data of fossil spore–pollen assemblages, to estimate species diversity relative to sample size. We use rarefaction because the count sizes for the samples slightly differed. Differential count sizes will bias any estimate of within-sample diversity as larger count sizes correspond to greater richness within a sample. Rarefaction allowed us to down-sample those larger samples until they are the same size as the smallest sample, making fair comparisons between incomplete samples.
Data collection	Samples were collected from the shallow-marine Río Turbio Formation in southern Patagonia.
Timing and spatial scale	Mid-late Eocene (42-33.5 Myr) from southern Patagonia.
Data exclusions	We excluded some samples as they contained no (or very few) palynomorphs.
Reproducibility	All matrices are provided to reproduce our analyses.
Randomization	Random samples (drops with microscopic spores and pollen grains to prepare slides with no allocation of any kind).
Blinding	Blinding was not relevant because we work with microscopic specimens and we cannot observe them while we sample them.

Did the study involve field work? ☒ Yes ☐ No

Field work, collection and transport

Field conditions	The current climatic conditions do not affect (by any means) the results of our analysis.
Location	Río Turbio, Santa Cruz Province, Southernmost South America 51°32'44.8"S 72°20'37.9"W
Access & import/export	All permits from the Santa Cruz province authorities were obtained beforehand to conduct fieldworks (see below).
Disturbance	No disturbances were caused during the fieldworks.

Reporting for specific materials, systems and methods

We require information from authors about some types of materials, experimental systems and methods used in many studies. Here, indicate whether each material, system or method listed is relevant to your study. If you are not sure if a list item applies to your research, read the appropriate section before selecting a response.

Materials & experimental systems

n/a	Involved in the study	
☒	☐	Antibodies
☒	☐	Eukaryotic cell lines
☐	☒	Palaeontology and archaeology
☒	☐	Animals and other organisms
☒	☐	Human research participants
☒	☐	Clinical data
☒	☐	Dual use research of concern

Methods

n/a	Involved in the study	
☒	☐	ChIP-seq
☒	☐	Flow cytometry
☒	☐	MRI-based neuroimaging

Palaeontology and Archaeology

Specimen provenance	We obtained permits from the "Dirección de Patrimonio Cultural" (Santa Cruz Province) to conduct fieldwork in the province under the Provincial Law Number 3137. The permits was issued the 17 December 2013.
Specimen deposition	The specimens are housed at the Museo "Padre Jesús Molina" under the catalogue numbers 21647–21699, prefixed MPM-PB.
Dating methods	We used biostratigraphy to date our sediments, including the use of key species of dinoflagellates; this is specified in the supplementary section or our work.

☒ Tick this box to confirm that the raw and calibrated dates are available in the paper or in Supplementary Information.

Ethics oversight	No ethical approval was required as we are working with fossil specimens.

Note that full information on the approval of the study protocol must also be provided in the manuscript.
